# The prognostic significance of preoperative left ventricular diastolic dysfunction and left atrial enlargement on acute coronary syndrome in kidney transplantation

**DOI:** 10.18632/oncotarget.16862

**Published:** 2017-04-05

**Authors:** Jin Ho Hwang, Jun-Bean Park, Yong-Jin Kim, Jung Nam An, Jaeseok Yang, Curie Ahn, In Mok Jung, Chun Soo Lim, Yon Su Kim, Young Hoon Kim, Jung Pyo Lee

**Affiliations:** ^1^ Nephrology Department of Internal Medicine, Chung-Ang University Hospital, Seoul, Korea; ^2^ Cardiology Department of Internal Medicine, Seoul National University College of Medicine, Seoul, Korea; ^3^ Nephrology Department of Internal Medicine, Seoul National University Boramae Medical Center, Seoul, Korea; ^4^ Department of Critical Care Medicine, Seoul National University Boramae Medical Center, Seoul, Korea; ^5^ Transplantation Center, Seoul National University Hospital, Seoul, Korea; ^6^ Nephrology Department of Internal Medicine, Seoul National University College of Medicine, Seoul, Korea; ^7^ Department of Surgery, Seoul National University Boramae Medical Center, Seoul, Korea; ^8^ Department of Surgery, Asan Medical Center and University of Ulsan College of Medicine, Seoul, Korea

**Keywords:** acute coronary syndrome, cardiovascular disease, transthoracic echocardiography, kidney transplantation, renal transplantation

## Abstract

Echocardiography is commonly performed as a screening test to evaluate perioperative risks before kidney transplantation. However, only limited data are available on echocardiographic parameters of left ventricular diastolic dysfunction (LVDD) and left atrial enlargement (LAE) on acute coronary syndrome and mortality in kidney transplant recipients. We reviewed 2779 adult recipients who underwent pretransplant echocardiography from 1997 to 2012. We divided the patients into two and four groups by two categories: LVDD grades 0–1 vs. 2–3, and left atrial size quartile groups. During a mean follow-up of 4.5 years, acute coronary syndrome occurred in 89 (3.2%) patients. The recipients with LVDD grades 2–3 (*P* = 0.005 for non-fatal, *P* = 0.02 for fatal/non-fatal) and LAE (*P* = 0.001 for non-fatal, *P* = 0.03 for fatal/non-fatal) had a higher incidence of acute coronary syndrome after kidney transplantation. All-cause mortality did not differ significantly between the groups. In a multivariate analysis, LVDD of grades 2–3 (hazard ratio 2.98, 95% confidence interval 1.535–5.787; *P* = 0.001), and LAE (hazard ratio 1.052, 95% confidence interval 1.006–1.101; *P* = 0.03) were independently associated with non-fatal acute coronary syndrome. In patients who are kidney transplant candidates, pretransplant LVDD and LAE were independently associated with a higher incidence of acute coronary syndrome after kidney transplantation.

## INTRODUCTION

Although kidney transplant (KT) recipients have demonstrated improved survival compared with patients undergoing dialysis, cardiovascular (CV) mortality is the leading cause of death following KT, accounting for 40–55% of all deaths [1, 2]. Identifying patients who are at high risk for CV disease could be important for offering appropriate management before KT.

Several baseline echocardiographic abnormalities have been found to have prognostic significance in patients with ESRD [3–5], but only limited data on echocardiographic prognostic factors are available in KT recipients [6–9]. Especially, there has been a lack of relevant research of the effect of pre-KT LVDD and left atrial enlargement (LAE) on posttransplant acute coronary syndrome (ACS).

In the general population, an increase in left atrial volume index (LAVI) and E wave over tissue-Doppler imaging of the E wave (E/E’) ratio is associated with elevated left ventricular (LV) filling pressures and significant diastolic dysfunction [10]. In KT recipients, there have been observed a significant reduction in left ventricular diastolic dysfunction (LVDD) rates after KT, and it may be caused by resolved occult volume overload [11, 12]. Despite the potential benefits of KT on cardiac function, one study suggested that pre-KT left ventricular hypertrophy (LVH), ventricular dilatation, and systolic dysfunction were associated with higher all-cause mortality and CV mortality after KT [7]. In another study, age, LV end-systolic diameter, maximal wall thickness, and mitral annular calcification were proposed as independent predictors of mortality after KT [8].

The 2012 AHA Scientific Statement recommended considering noninvasive stress testing in KT candidates with no active cardiac conditions, based on the presence of multiple CAD risk factors [13]. Non-stress echocardiography has been routinely conducted in Korea for assessing baseline heart function before KT. In contrast, non-invasive stress tests were not widely performed in KT candidates before 2012.

The aim of this study was to identify the prognostic effect of pretransplant conventional echocardiographic findings with LVDD and LAE for predicting outcomes of acute coronary syndrome (ACS) and mortality in KT recipients.

## RESULTS

### Baseline data by LVDD and outcomes

The baseline characteristics of 2779 enrolled patients are described in Table 1. In higher-grade LVDD, age at transplantation was younger and the proportion of men and smokers was higher. The mean age of patients at KT was 41.7 years, and 59.6% were men. During a mean follow-up of 4.5 years, non-fatal ACS and composite of fatal/non-fatal ACS occurred in 49 (1.8%) and 89 (3.2%) patients respectively, and 116 (6.2%) died.

**Table 1:** Baseline characteristics of the study subjects

	Total (*N* = 2779)	LVDD grade–based group		*P*-value	Total (*N* = 2727)	LA size–based group				*P*-value
		LVDD grades 0–1 (*n* = 2491)	LVDD grades 2–3 (*n* = 288)			Group 1 LA size< 36 mm (*n* = 747 )	Group 2 36 ≤ LA size < 40 mm (*n* = 739)	Group 3 40 ≤ LA size < 44 mm (*n* = 609)	Group 4 LA size ≥ 44 mm (*n* = 632)	
Age at transplantation (years)^a^	41.7 ± 11.6	41.9 ± 11.7	40.5 ± 10.8	0.05	42.0 ± 11.3	39.0 ± 11.0	41.6 ± 10.9	43.6 ± 11.2	44.5 ± 11.3	< 0.001^d^
Recipient’s sex (male, %)	59.6	58.0	72.9	< 0.001	59.4	46.6	57.0	63.5	73.6	< 0.001
BMI (kg/m^2^)^a^	22.9 ± 11.6	23.0 ± 12.2	22.5 ± 2.9	0.51	23.0 ± 11.7	21.8 ± 10.2	23.2 ± 13.5	23.1 ± 8.2	24.0 ± 13.6	0.008^e^
Current smoker (%)	9.5	9.0	14.6	< 0.001	9.6	6.0	8.8	11.0	13.3	< 0.001
Comorbidities (%)										
Hypertension	82.4	82.1	85.4	0.19	82.8	75.9	81.5	87.7	88.0	< 0.001
Diabetes mellitus	19.3	18.8	23.6	0.06	19.6	12.7	18.3	24.1	25.0	< 0.001
Vascular disease^b^	6.2	6.0	7.6	0.30	6.2	4.3	6.1	6.4	8.5	0.01
Dyslipidemia	55.3	53.9	71.8	0.04	57.3	54.0	59.7	53.7	61.6	0.52
Dialysis before KT	83.8	83.6	86.6	0.63	83.7	82.7	82.7	84.2	85.5	0.77
HD	56.0	56.2	55.4		56.5	55.5	56.5	58.4	55.5	
PD	24.3	23.9	27.9		23.9	23.4	23.5	22.9	25.9	
Modality conversion (HD→PD or PD→HD)	3.5	3.5	3.3		3.3	3.8	2.7	2.9	4.1	
Recipient CMV IgG (+) (%)	57.3	57.6	54.9	0.38	58.2	61.3	58.7	58.8	53.5	0.03
Dialysis duration (months)^a^	33.2 ± 40.2	34.3 ± 13.2	31.3 ± 37.2	0.38	33.0 ± 40.3	31.4 ± 38.1	34.0 ± 42.5	31.2 ± 39.2	41.3 ± 1.7	0.16
Intact PTH (pg/mL)	229.6 ± 252.6	228.9 ± 246.6	236.0 ± 305.0	0.90	229.0 ± 255.3	277.9 ± 351.5	192.4 ± 166.0	263.3 ± 284.1	188.9 ± 181.0	0.15
Donor’s age (years)^a^	39.3 ± 12.1	39.4 ± 12.2	38.2 ± 11.3	0.27	39.2 ± 12.1	39.6 ± 12.6	39.5 ± 11.8	38.9 ± 12.3	38.7 ± 11.8	0.44
Donor’s sex (male, %)	58.0	57.9	59.0	0.75	58.2	60.6	55.7	57.2	59.3	0.26
Deceased donor (%)	24.3	24.7	20.8	0.19	23.9	23.4	23.8	21.5	26.8	0.17
Donor CMV IgG (+) (%)	60.3	60.6	57.3	0.28	61.1	62.7	58.9	64.9	58.2	0.04
Steroid maintenance strategy (%)	91.8	91.3	97.0	0.50	91.8	92.6	90.9	89.6	95.4	0.58
CNI (CsA:Tacrolimus, %)	50.7:49.0	50.2:49.4	54.5:45.5	0.49	50.8:48.9	48.8:50.6	49.7:50.2	51.2:48.8	54.3:45.5	0.29
Antimetabolites (Aza:MMF, %)	19.9:77.0	19.2:77.5	26.3:72.8	0.03	20.1:76.9	19.9:77.9	19.0:75.8	19.3:78.5	22.3:75.4	0.01
CMV disease (%)	5.7	5.8	4.5	0.54	5.7	7.2	5.2	5.3	5.0	0.36
Pretransplant echocardiographic findings											
LA size (mm)^a^	39.2 ± 6.3		38.2 ± 5.7	47.2 ± 5.2	< 0.001	39.2 ± 6.3	31.8 ± 2.9	37.6 ± 1.1	41.3 ± 1.1	47.6 ± 3.5	< 0.001^f^
Grades 2–3 LVDD (%)	—	—	—	—	10.5	0.9	1.2	5.4	37.5	< 0.001
LVIDs (mm)^a^	33.1 ± 6.1	32.6 ± 5.7	37.7 ± 7.3	< 0.001	33.1 ± 6.1	31.8 ± 2.9	37.6 ± 1.1	41.3 ± 1.1	47.6 ± 3.5	< 0.001^f^
LVIDd (mm)^a^	51.8 ± 6.2	51.2 ± 5.9	56.8 ± 6.2	< 0.001	51.8 ± 6.2	48.1 ± 5.2	51.2 ± 4.9	52.8 ± 5.2	56.2 ± 6.3	< 0.001^f^
IVSd (mm)^a^	10.7 ± 2.1	10.6 ± 2.1	11.9 ± 2.1	< 0.001	10.7 ± 2.1	9.6 ± 1.8	10.5 ± 1.7	11.1 ± 2.0	12.1 ± 2.1	< 0.001^f^
LV ejection fraction (EF, mean of %)^a^	60.4 ± 7.4	60.7 ± 7.0	57.6 ± 10.0	< 0.001	60.4 ± 7.4	60.8 ± 6.5	61.3 ± 6.4	60.9 ± 6.7	58.2 ± 9.5	< 0.001^g^
LVEF < 50% (%)	6.9	5.6	18.2	< 0.001	6.8	4.4	4.5	5.3	13.9	<0.001
LV mass index (g/m^2^)	121.6 ± 35.9	118.0 ± 34.3	155.0 ± 32.9	< 0.001	124.0 ± 34.4	100.4 ± 25.8	124.6 ± 38.6	127.5 ± 29.6	152.7 ± 35.8	< 0.001^i^
Severe LVH (%)^c^	65.9	63.1	90.3	< 0.001	66.9	39.2	67.4	76.2	89.9	< 0.001
E/A ratio^a^	1.08 ± 0.42	1.02 ± 0.36	1.57 ± 0.59	< 0.001	1.08 ± 0.42	1.10± 0.39	1.05 ± 0.36	1.02 ± 0.36	1.14 ± 0.53	< 0.001^i^
E/E’^a^	12.4 ± 5.0	11.4 ± 4.0	18.9 ± 6.1	< 0.001	12.4 ± 5.0	9.8 ± 3.3	11.7 ± 3.9	12.6 ± 4.1	16.2 ± 6.2	< 0.001^i^
MVDT (msec)^a^	208 ± 61	210 ± 61	191 ± 61	0.06	212 ± 59	205 ± 62	206 ± 48	213 ± 53	224 ± 71	0.06
Systolic PAP (mmHg)^a^	29.9 ± 8.7	28.6 ± 6.8	39.4 ± 13.2	< 0.001	29.9 ± 8.6	26.1 ± 5.0	28.0 ± 5.6	30.1 ± 8.5	35.6 ± 11.0	< 0.001^f^

^a^Data are expressed as the mean ± SD.

^b^Vascular disease included cardiovascular, cerebrovascular, and peripheral vessel diseases.

^c^Severe LVH was defined as LVMI of > 120 g/m^2^ for women and > 150 g/m^2^ for men.

LVM (g) = 1.05[(LVEDD + IVS + PW)^3^ − LVEDD^3^]

LVMI (g/m^2^) = Left ventricular mass/body surface area

^d^*P* < 0.05 at post-hoc analysis between all the groups except for group 3 and 4.

^e^*P* < 0.05 at post-hoc analysis between group 1 and 4.

^f^*P* < 0.05 at post-hoc analysis between all the groups.

^g^*P* < 0.05 at post-hoc analysis between the each of group 1, 2, 3 and group 4.

^h^*P* < 0.05 at post-hoc analysis between group 1 and 3, 2 and 4, and 3 and 4.

^i^*P* < 0.05 at post-hoc analysis between all the groups except for group 2 and 3.

LVDD: left ventricular diastolic dysfunction; LA: left atrium; BMI: body mass index; KT: kidney transplantation; HD: hemodialysis; PD: peritoneal dialysis; CMV IgG: cytomegalovirus immunoglobulin G; PTH: parathyroid hormone; CNI: calcineurin inhibitor; CsA: cyclosporine A; Aza: azathioprine; MMF: mycophenolate mofetil; LVIDs: left ventricular internal dimension in systole; LVIDd: left ventricular internal dimension in diastole; IVSd: interventricular septum in diastole; LVH: left ventricular hypertrophy; MVDT: mitral valve deceleration time; PAP: pulmonary artery pressure.

Comorbidities, dialysis and donor factors did not differ between the two groups. Preemptive KT was performed in 16.2% of patients, and hemodialysis was performed before KT in 55% of patients. Higher-grade LVDD was associated with more cases of dilated LA, LV, and decreased LV systolic function.

**Figure 1:**
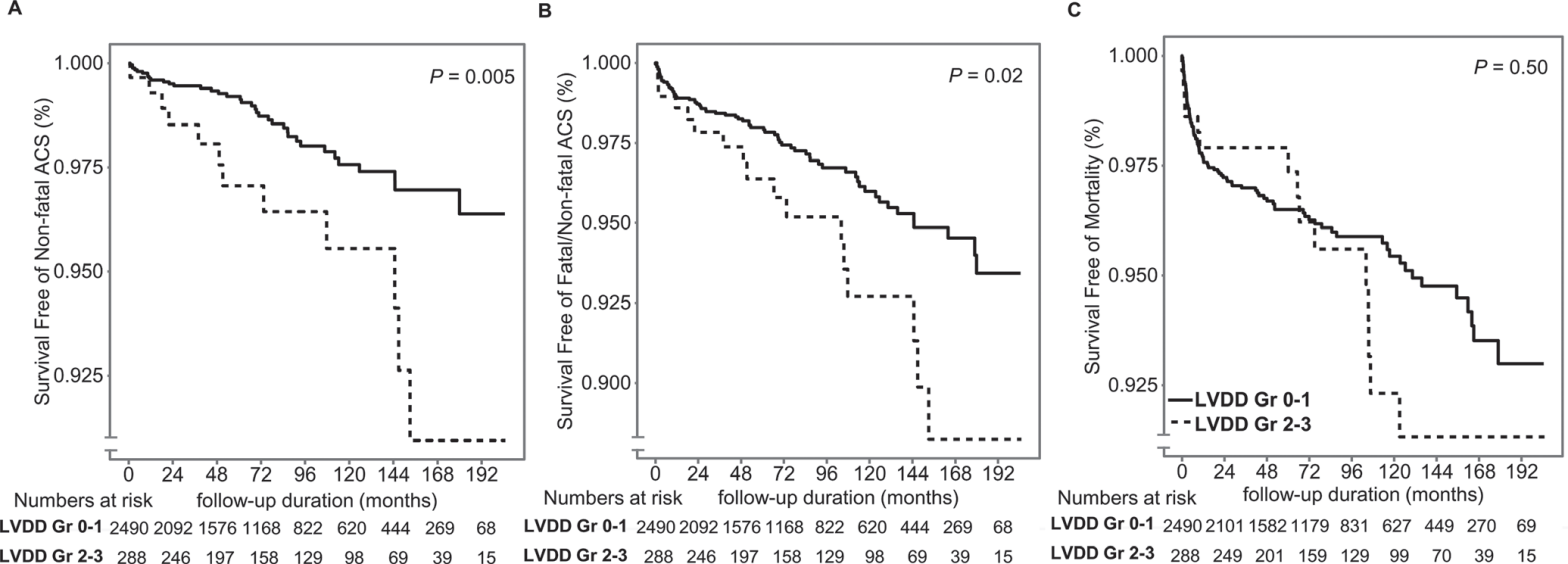
Kaplan–Meier curves for posttransplant occurrence of ACS and all-cause mortality in the LVDD grade–based group (**A, B**) Patients with LVDD grades 2–3 showed worse ACS outcomes than the patient group with LVDD grades 0–1 (*P* = 0.005 for non-fatal, *P* = 0.02 for fatal/non-fatal ACS). (**C**) All-cause mortality did not differ between the two groups (*P* = 0.50).

ACS occurred significantly more often in the group diagnosed with LVDD grades 2–3 (*P* = 0.005 for non-fatal ACS, *P* = 0.02 for fatal/non-fatal ACS). No meaningful difference was seen in all-cause mortality (*P* = 0.50) (Figure 1).

### Baseline data by LAE and outcomes

The baseline characteristics of 2727 patients are described in Table 1. Larger size LA was associated with more dilated LV, LVH, and decreased LV systolic function than smaller size LA (Table 1).

**Figure 2:**
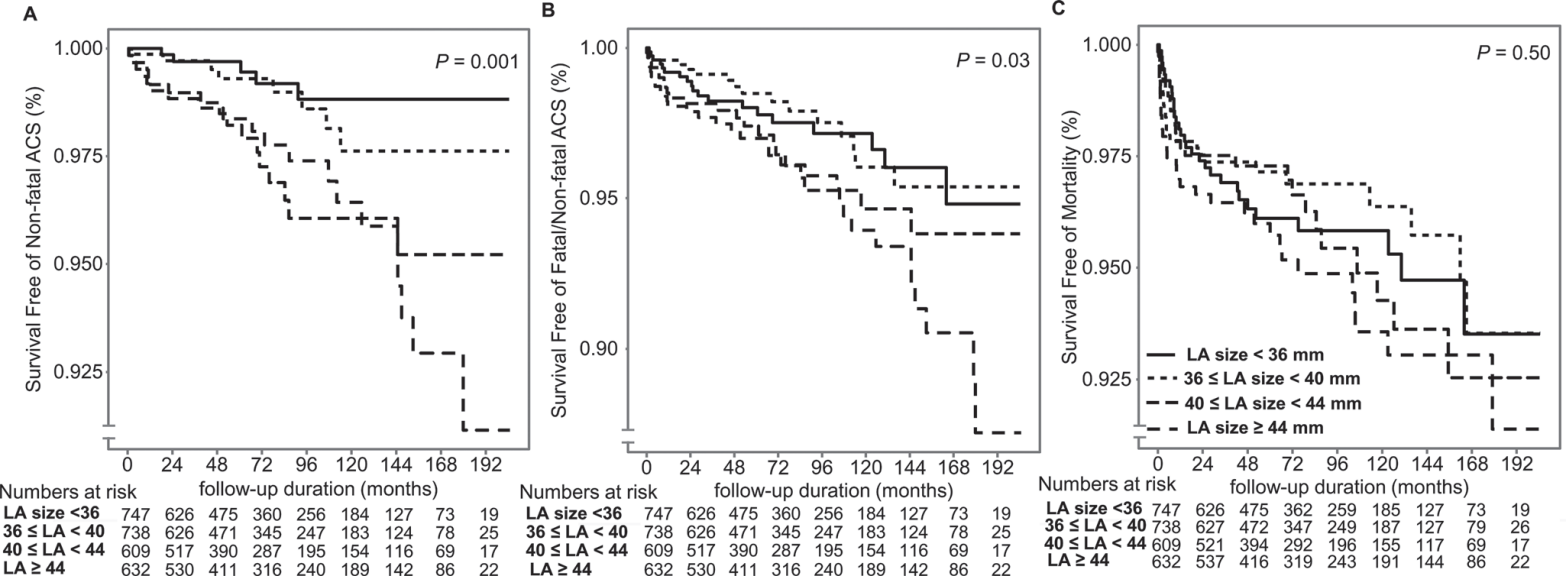
Kaplan–Meier curves for post-KT occurrence of ACS and all-cause mortality in the LA size–based group (**A, B**) Patients with larger LA size showed worse ACS outcomes than patients with smaller LA size (*P* = 0.001 for non-fatal, *P* = 0.03 for fatal/non-fatal ACS). (**C**) All-cause mortality did not differ between the two groups (*P* = 0.50).

**Figure 3:**
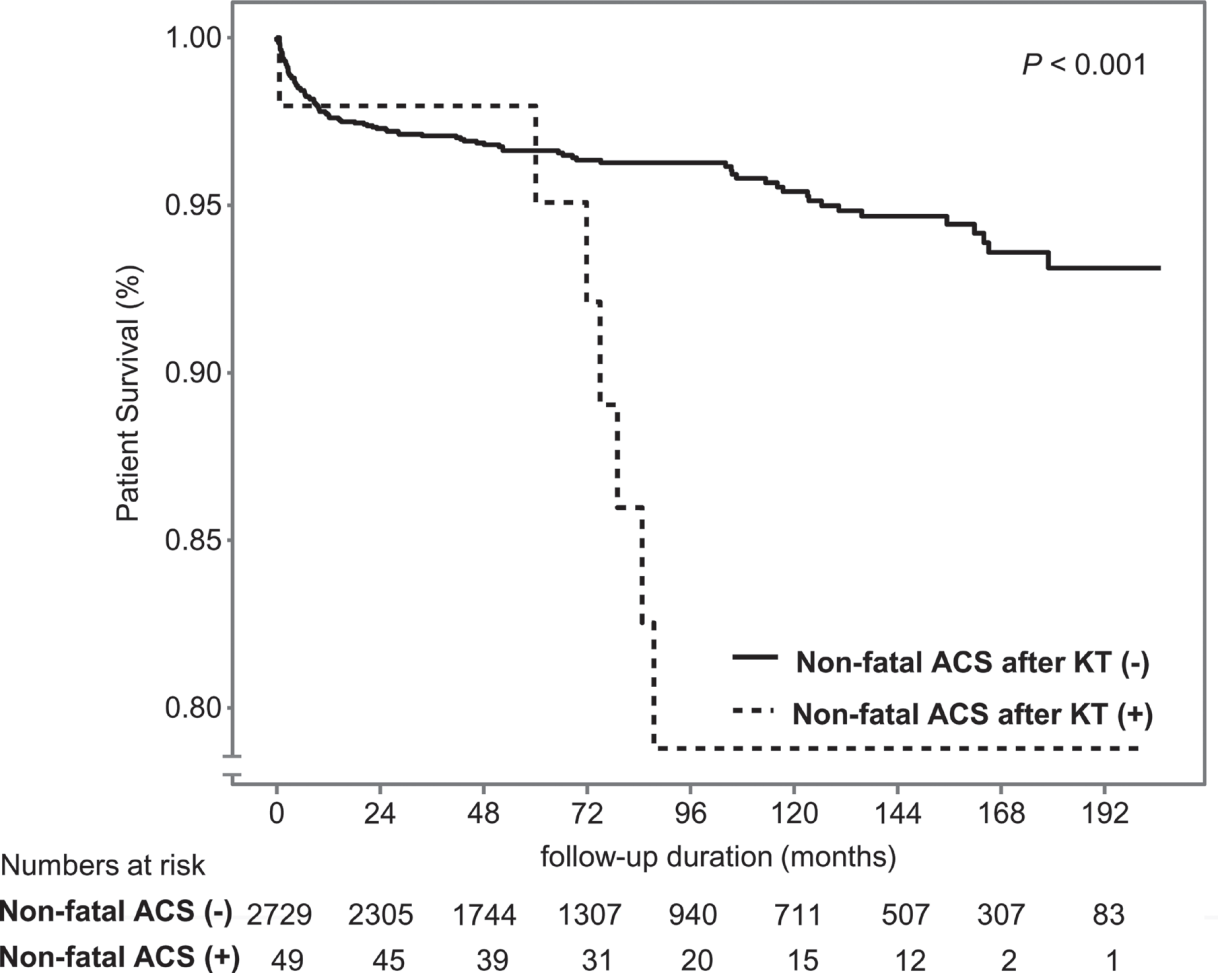
Kaplan–Meier curve for all-cause mortality by non-fatal ACS after KT (*P* < 0.001)

When we analyzed patients by LA size, ACS occurred more frequently in the subgroup with larger LA size, similar to previous results (*P* = 0.001 for non-fatal ACS, *P* = 0.03 for fatal/non-fatal ACS). No meaningful difference was seen in all-cause mortality (*P* = 0.50), as in the LVDD group (Figure 2). The patients who experienced non-fatal ACS showed increased all-cause mortality than others especially after 5 years of KT (*P* < 0.001, Figure 3).

### Subgroup analysis of LVDD and LAE

**Figure 4:**
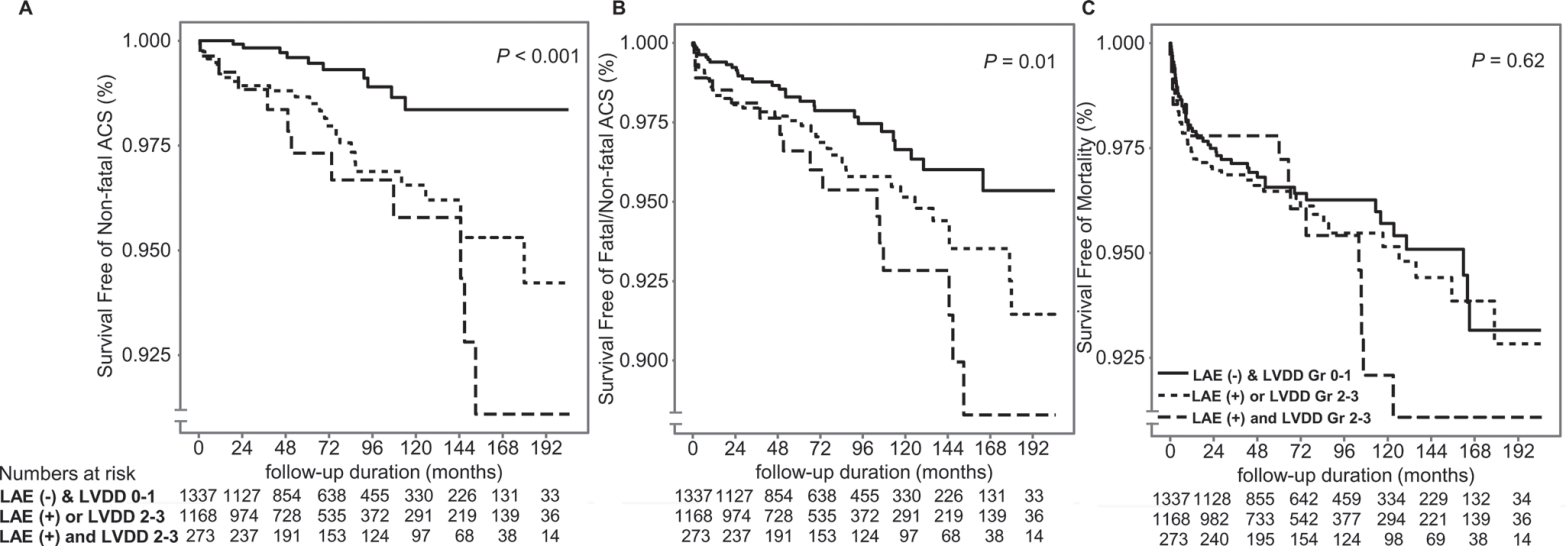
Kaplan–Meier curves for post-KT occurrence of ACS, GF, and all-cause mortality in the LVDD grade– and median LA size based groups (**A, B**) In patients who had both LVDD grades 2–3 and LAE, ACS occurred significantly more often than in patients who were diagnosed with LVDD or LAE alone (*P* = 0.001 for non-fatal ACS, *P* = 0.01 for fatal/non-fatal ACS). (**C**) Results for all-cause mortality were statistically insignificant overall (*P* = 0.62).

In the patients who had both LVDD grades 2–3 and LAE, ACS occurred significantly more often than others (*P* < 0.001 for non-fatal ACS, *P* = 0.01 for fatal/non-fatal ACS). The result for mortality (*P* = 0.62) was statistically insignificant overall (Figure 4), though the mortality was higher in patients who had both LVDD grades 2–3 and LAE, specifically in the population who was followed up for > 5 years (*P* = 0.008, data not shown).

We further analyzed the outcomes by comparing preemptive KT and long-term dialysis before KT. In patients who underwent preemptive KT, ACS and mortality did not differ according to LVDD and LA size based groups, but in patients who underwent long-term dialysis before KT, occurrences of non-fatal ACS (*P* = 0.001) and fatal/non-fatal ACS (*P* = 0.005) were significantly higher in LVDD grades 2–3 group (Supplementary Figures 1 and 2). When we analyzed patients who underwent long-term dialysis before KT, a diagnosis of LVDD grades 2–3 was associated with a higher incidence of non-fatal ACS (*P* = 0.006) and fatal/non-fatal ACS (*P* = 0.005) only in patients who underwent hemodialysis before KT. All-cause mortality (*P* = 0.08) did not show significant differences between groups according to dialysis modality (Supplementary Figure 3).

In aspects of concomitant LV systolic dysfunction, non-fatal ACS occurred more in patients who had both LVDD grades 2–3 and LAE (*P* = 0.001) when LVEF was ≥ 50%. None of the outcomes were significantly different by the LVDD grades-based groups, LA size-based groups, or LVDD and LA composite groups in LVEF < 50% (data not shown).

### Factors affecting the occurrence of ACS

We conducted multivariate analysis for the occurrence of ACS (Table 2). When we adjusted for age, sex, hypertension, DM, dyslipidemia, smoking, previous history of IHD or previous history of vascular disease, and aortic regurgitation (AR), we found that increased age (*P* = 0.001), hypertension (*P* = 0.04), DM (*P* = 0.02), previous history of CV events (*P* = 0.001), and LVDD grades 2–3 (HR 2.980, 95% CI 1.535–5.787; *P* = 0.001) were associated with non-fatal ACS in KT recipients. When we analyzed the LAE size, after adjusting for the same variables (except for LVDD grade), we also found a significantly higher incidence of ACS (HR 1.052, 95% CI 1.006–1.101; *P* = 0.03)(Table 2). In these models, LVDD grades 2–3 were associated with a threefold increased relative risk of non-fatal ACS, and a 1-mm increase in LA size was associated with a 5.2% increase in relative risk of non-fatal ACS (*P* = 0.001). However, neither LVDD nor LAE was significantly associated with all-cause mortality in a multivariate analysis.

**Table 2:** Factors associated with the occurrence of fatal/non-fatal ACS after KT

	Non-fatal ACS						Fatal/non-fatal ACS					
	Univariate			Multivariatea			Univariate			Multivariate^a^		
	HR	95% CI	*P*-value	HR	95% CI	*P*-value	HR	95% CI	*P*-value	HR	95% CI	*P*-value
Age (per year)	1.097	1.066–1.129	< 0.001	1.099	1.063–1.135	< 0.001	1.058	1.038–1.079	< 0.001	1.054	1.032–1.077	< 0.001
Female	0.527	0.278–0.998	0.05	1.196	0.619–2.314	0.59	0.599	0.377–0.952	0.03	1.288	0.806–2.057	0.29
Hypertension	10.422	1.435–75.686	0.02	7.747	1.057–56.807	0.04	2.199	1.057–4.577	0.04	1.839	0.879–3.849	0.11
Diabetes mellitus	5.342	3.018–9.457	< 0.001	2.199	1.156–4.183	0.02	3.117	2.022–4.803	< 0.001	1.889	1.171–3.048	0.009
Dyslipidemia	2.155	0.756–6.141	0.15	1.058	1.002–1.117	0.83	1.525	0.598–3.890	0.38	0.972	0.373–2.532	0.95
Smoking	1.878	0.871–4.048	0.11	1.125	0.511–2.476	0.77	1.209	0.618–2.363	0.58	0.834	0.424–1.640	0.60
Previous history of IHD	11.095	5.776–21.310	< 0.001	5.323	2.130–13.301	< 0.001	6.668	3.778–11.766	< 0.001	4.312	2.445–7.604	< 0.001
Previous history of vascular disease	8.000	4.310–14.850	< 0.001	3.256	1.712–6.583	< 0.001	5.193	3.099–8.700	< 0.001	2.900	1.675–5.022	< 0.001
Donor sex (female)	1.220	0.685–2.175	0.50	—	—	—	0.883	0.570–1.368	0.58	—	—	—
Donor age	1.001	0.995–1.007	0.79	—	—	—	1.000	0.994–1.006	0.99	—	—	—
HD	1.853	0.641–5.353	0.26	—	—	—	1.158	0.545–2.459	0.70	—	—	—
Deceased donor	1.683	0.850–3.333	0.14	—	—	—	1.463	0.881–2.431	0.14	—	—	—
NODAT	1.804	0.805–4.041	0.15	—	—	—	2.663	1.331–5.330	0.006	2.033	0.936–4.416	0.07
Intact PTH	1.000	0.997–1.003	0.94	—	—	—	1.000	0.997–1.003	0.94	—	—	—
LA diameter (per 1 mm)^a^	1.101	1.056–1.148	< 0.001	1.052	1.006–1.101	0.03	1.064	1.030–1.099	< 0.001	1.033	1.002–1.068	0.05
LV ejection fraction	0.996	0.959–1.033	0.82	—	—	—	0.989	0.963–1.016	0.42	—	—	—
Diastolic dysfunction (grades 2–3)^a^	2.882	1.486–5.593	0.002	2.980	1.535–5.787	0.001	2.108	1.224–3.628	0.007	1.908	1.122–3.246	0.02
Valvular disease												
MR (grade 3)	2.365	0.308–18.188	0.41	—	—	—	2.935	0.668–12.901	0.15	—	—	—
TR (grade 3)	3.526	0.433–28.715	0.24	—	—	—	2.200	0.275–17.600	0.46	—	—	—
AR (grade 2)	5.671	1.279–25.150	0.02	3.570	0.831–15.331	0.09	3.139	0.718–13.729	0.13	2.052	0.492–8.536	0.32
CMV disease after transplantation	1.864	0.557–6.238	0.31	—	—	—	1.090	0.389–3.052	0.87	—	—	—

Data were analyzed by using the Cox regression, Enter method in the multivariate analysis.

^a^Age, sex, hypertension, DM, dyslipidemia, smoking, previous history of IHD, and AR were used as covariates with one of LA diameter or diastolic dysfunction.

IHD: ischaemic heart disease; HD: hemodialysis; NODAT: new-onset diabetes after transplant; LA: left atrium; LV: left ventricle; MR: mitral regurgitation; TR: tricuspid regurgitation; AR: aortic regurgitation; CMV: cytomegalovirus.

Incrementally, the predictive values of LVDD and LA size showed significantly superior powers of discrimination for ACS (Table 3). After adjusting for traditional risk factors, such as age, hypertension, DM, and smoking, including LVDD increased area under the receiver operating characteristic (ROC) curve (AUC) from 0.721 to 0.762 (*P* = 0.04) for predicting non-fatal ACS occurrence. The net reclassification improvement (NRI) and integrated discrimination improvement (IDI) also showed significant improvements in discrimination (NRI = 0.486; *P* = 0.001, IDI = 0.009; *P* = 0.02). LA size showed a similar, but less significant improvement (NRI = 0.346; *P* = 0.02, IDI = 0.008; *P* = 0.02)(Table 3).

**Table 3:** Incremental value of LVDD or LA size over traditional risk factors for predicting ACS after KT

	AUC		Category-free NRI		IDI	
	Value	*P*-value	Value	*P*-value	Value	*P*-value
Traditional risk factors^a^	0.721	—	—	—	—	—
+ LVDD	0.762	0.04	0.486	< 0.001	0.009	0.02
+ LA size	0.775	0.08	0.346	0.02	0.008	0.02

Data were analyzed using area under the receiver operating characteristic curve (AUC), net reclassification improvement (NRI), and integrated discrimination improvement (IDI) with R software.

^a^Results of conventional risk factors (age, DM, hypertension, smoking) were taken as reference values for analyses.

LVDD: left ventricular diastolic dysfunction; LA: left atrium; ACS: acute coronary syndrome; AUC: area under the receiver operating characteristic curve; NRI: net reclassification improvement; IDI: integrated discrimination improvement.

## DISCUSSION

Interest in CV outcomes after KT is increasing because CV disease is the leading cause of death and allograft loss in KT patients [14]. In this study of 2779 KT recipients, higher-grade LVDD was associated with more dilated LA, LV, and decreased LV systolic function. We also reported that pretransplant LVDD grades 2–3 and LAE were independently associated with a higher incidence of post-KT ACS, after multivariate analysis that adjusted for traditional risk factors.

The LVDD is frequently observed in ESRD patients. The association between LVDD and the occurrence of CAD had not been previously established in both CKD and normal renal function patients, although the association with heart failure and mortality had been established [15, 16]. A recent prospective cohort study reported that an increase in E/E’ or LAVI was an independent risk factor for CV events in incident ESRD patients with preserved LV systolic function [17]. Unlike our study, the recent study included arrhythmia as well as coronary artery, cerebrovascular, and peripheral vascular disease in defining cardiovascular events. The study demonstrated that LVDD is closely related to the increase in LVMI and the association of LVDD with CAD by exemplifying several studies in which increased LVMI was associated with a higher incidence of CAD [17]. Our study also showed the marked increase of LVMI in LVDD grades 2–3 group than LVDD grades 0–1 group. Patients with LVDD were also known to have increased collagen levels in the myocardium [18]. This factor might be related to poor myocardial perfusion and increased CAD in patients with high-grade LVDD. Until now, there is little evidence to date of the association and mechanism of LVDD and CAD. This study is meaningful because it suggested that LVDD before transplantation is an independent factor in increasing post-transplant CAD occurrence.

LAE has been shown to indicate poor prognosis, not only in the general population [10, 19] but also in ESRD patients [5, 6, 20, 21], and KT recipients [7, 9]. In addition, it has been proposed that echocardiographic findings of LAE are useful markers of significant hemodynamic changes, which are in turn related to angiographically confirmed CAD [22–24]. LAE is a known manifestation of cardiac target-organ damage in the presence of established hypertension and increased LVM by enhancing cardiac oxygen demand and impairing LV filling and contractility [23, 24]. In our subgroup analysis, the patients who had both higher-grade LVDD and LAE had poorer CV outcomes than other patients. LAE has been suggested as a marker of LVDD severity and duration [10, 21], and it can lead to unfavorable outcomes related to irreversible functional or structural changes in the heart. Patients who were diagnosed with LAE without LVDD might have had atrial arrhythmia or mitral valve disease whose clinical implications are different.

In a multivariate analysis, neither higher-grade LVDD nor LAE before KT was significantly associated with post-KT all-cause mortality, although a previous study reported such an association [25]. The follow-up duration might have been too short to analyze mortality in this population. In addition, the mortality could have been affected by multifactorial components besides heart function, and the effect of LVDD and LAE might not have been sufficient to influence patient outcomes. Moreover, the parameters of LVDD and LAE are load-dependent and usually predict mortality mainly in patients with heart failure [26].

In another subgroup analysis, higher-grade LVDD in patients who underwent long term dialysis before KT showed poor CV outcomes, especially in patients with hemodialysis before KT. Volume overload pattern between hemodialysis and peritoneal dialysis is different and it varies Frank-Starling effects and the mechanism of worsening cardiac function over time [27,28]. When patients show high-grade LVDD or LAE, especially those undergoing hemodialysis, more attention to improving those parameters should be considered.

To date, only few reports have examined the association between pre-KT echocardiographic findings and KT outcomes [6–9], and these were limited by small sample size. To the best of our knowledge, this study was conducted with the largest number of KT recipients to date. It is also notable for enrolling patients in two major transplantation centers in South Korea. We evaluated patients using relatively simple parameters that are easily measured with conventional transthoracic echocardiography. In addition, we defined CV outcomes as ACS, which is more specific to IHD and clear than “major adverse cardiac event”.

Studies of CV disease frequently exclude chronic kidney disease or ESRD patients from enrollment. Our results are meaningful because we focused on ESRD patients who were awaiting KT and, after adjusting for all known risk factors, showed an independent association between easily determined echocardiographic findings (LVDD and LAE) before KT and CV disease outcomes after KT. To verify the consistency of our results, we conducted various statistical assessments of incremental predictive values.

This study has some limitations. First, it is a retrospective design, so LAVI was not routinely measured during echocardiography. LAVI is a more standardized method than linear LA size measurements for evaluating LA size in patients with a great variety of body sizes and should be preferred over linear dimensions [29]. Second, body habitus and gender are essential in categorizing LA size as normal or abnormal. To overcome these limitations, we evaluated the outcomes with LA size and/or LVDD, and divided the patients into four groups by LA size quartile. In addition, echocardiography was not performed at specific points between scheduled dialysis sessions, and there could have been significant differences in echocardiographic results before and after dialysis sessions. However, despite the limitations, our study could have significance per se because it would not be easy to perform randomized controlled studies with these subjects. Lastly, the population of this study was all Korean, so the results are not comparable with US or European cohorts. Korean KT recipients showed lower incidence of CV disease (including peripheral vascular disease, 2.4% at 5 year, and 11.4% at 12 year after KT) in former research [30], and the incidence of ACS in this study was also low. It might be because of low prevalence of DM, younger age, and/or ethnic disparity.

To further improve the outcomes of KT recipients, it is necessary to evaluate their modifiable risk factors associated with CV disease. Data from this study suggest that adverse CV outcomes may occur after KT in patients with pre-KT LVDD and LAE.

We have shown that the presence of higher-grade LVDD and LAE before KT is an independent predictor of posttransplant ACS. In addition, LVDD and LAE both showed significant superior discrimination power over traditional risk factors used to predict ACS. Further investigations are needed to assess whether improving those findings before KT would impact KT outcomes, and whether more active evaluation and treatment of CAD would be helpful for improving outcomes in these patients.

## MATERIALS AND METHODS

### Study design and patients

A total of 4650 patients who underwent KT at two major institutions for transplantation in South Korea (Seoul National University Hospital and Asan Medical Center) were screened. To evaluate the prognostic significance of pretransplant echocardiographic findings on outcomes in KT, researchers collected data from the 2779 adult recipients who had undergone pretransplant echocardiography from January 1997 to January 2012 and who had available data of LVDD or LA size. All patients were ≥ 15 years of age, and had pretransplant echocardiographic findings ≤ 1 year before KT. Basic clinical parameters were collected, such as age at the time of KT, sex, body mass index, comorbidities, dialysis modality/duration before KT, pretransplant echocardiographic findings and donor factors. This study was approved by the institutional review board (H-1409–086–609), and the need for informed consent was waived because this study used a retrospective design. All clinical investigations were conducted in accordance with the guidelines of the 2013 Declaration of Helsinki.

### Definitions and grouping

LVDD was diagnosed according to the recommendations of the European Study Group on Diastolic Heart Failure [31] and divided into four grades on the basis of diastolic function using E/E’ ratio, the E/A ratio, and the E wave deceleration time: 0 (normal); 1 (relaxation abnormality); 2 (pseudonormalization); and 3 (restrictive pattern) [32]. We divided the patients into two groups by LVDD grades: 0–1 vs. 2–3.

Meanwhile, patients were divided into four groups according to LA size quartiles: lowest quartile group is < 36 mm (group 1), second quartile group is 36 mm ≤ LA size < 40 mm (group 2), third quartile group is 40 mm ≤ LA size < 44 mm (group 3), and largest quartile group is ≥ 44 mm (group 4).

For subgroup analysis, patients were divided into three groups by both LA size (median value of total study population: ≤ 38 mm or > 38 mm) and LVDD groups (LVDD group 0–1 or 2–3).

LV systolic dysfunction was defined as ejection fraction < 50%. The ACS was defined as universal definition of ST elevation myocardial infarction (MI), non-ST elevation MI, and unstable angina [33].

### Primary and secondary objectives

The primary objective of this study was to evaluate whether pretransplant echocardiographic findings predicted the occurrence of ACS after KT. The secondary objective of this study was to evaluate all-cause mortality.

### Statistical analysis

Most analyses were performed using IBM SPSS Statistics software v21.0 (IBM Corp., NY, USA). Continuous variables were expressed as the mean±standard deviation and were compared by using Student’s *t*-test. For categorical variables, data were expressed as percentages and compared by using the Chi-square test. The Cox regression model was used to identify independent risk factors by calculating HR and 95% CI. Differences whose *P*-value was < 0.05 were considered statistically significant. Using R version 3.2.0 (R Foundation for Statistical Computing, Wien, Austria), we evaluated the predictive contribution of LVDD or LA size to ACS risk using AUC, NRI, and IDI. ACS-free survival rates and other event-free survival rates were calculated using the Kaplan-Meier method, and group comparison was performed by using the log-rank test.

## SUPPLEMENTARY MATERIALS FIGURES



## Abbreviations

ACS: acute coronary syndrome
AUC: area under the curve
CAD: coronary artery disease
CI: confidence interval
CV: cardiovascular
DM: diabetes mellitus
E/E’: E wave over tissue-Doppler imaging of the E wave
ESRD: end-stage renal disease
HR: hazard ratio
IDI: integrated discrimination improvement
KT: kidney transplant or kidney transplantation
LAE: left atrial enlargement
LAVI: left atrial volume index
LVDD: left ventricular diastolic dysfunction
LVH: left ventricular hypertrophy
LVMI: left ventricular mass index
NRI: net reclassification improvement
ROC: receiver operating characteristic
SD: standard deviation
