# Supplementary material for: The prognostic significance of preoperative left ventricular diastolic dysfunction and left atrial enlargement on acute coronary syndrome in kidney transplantation
Source: Oncotarget. 2017 Apr 5;8(41):71154–63. doi: 10.18632/oncotarget.16862 (PMC5642626; doi:10.18632/oncotarget.16862)
Supplement: Supplementary file 1 [file oncotarget-08-71154-s001.pdf]

The prognostic significance of preoperative left ventricular diastolic dysfunction and left atrial enlargement on acute coronary syndrome in kidney transplantation

Supplementary Materials

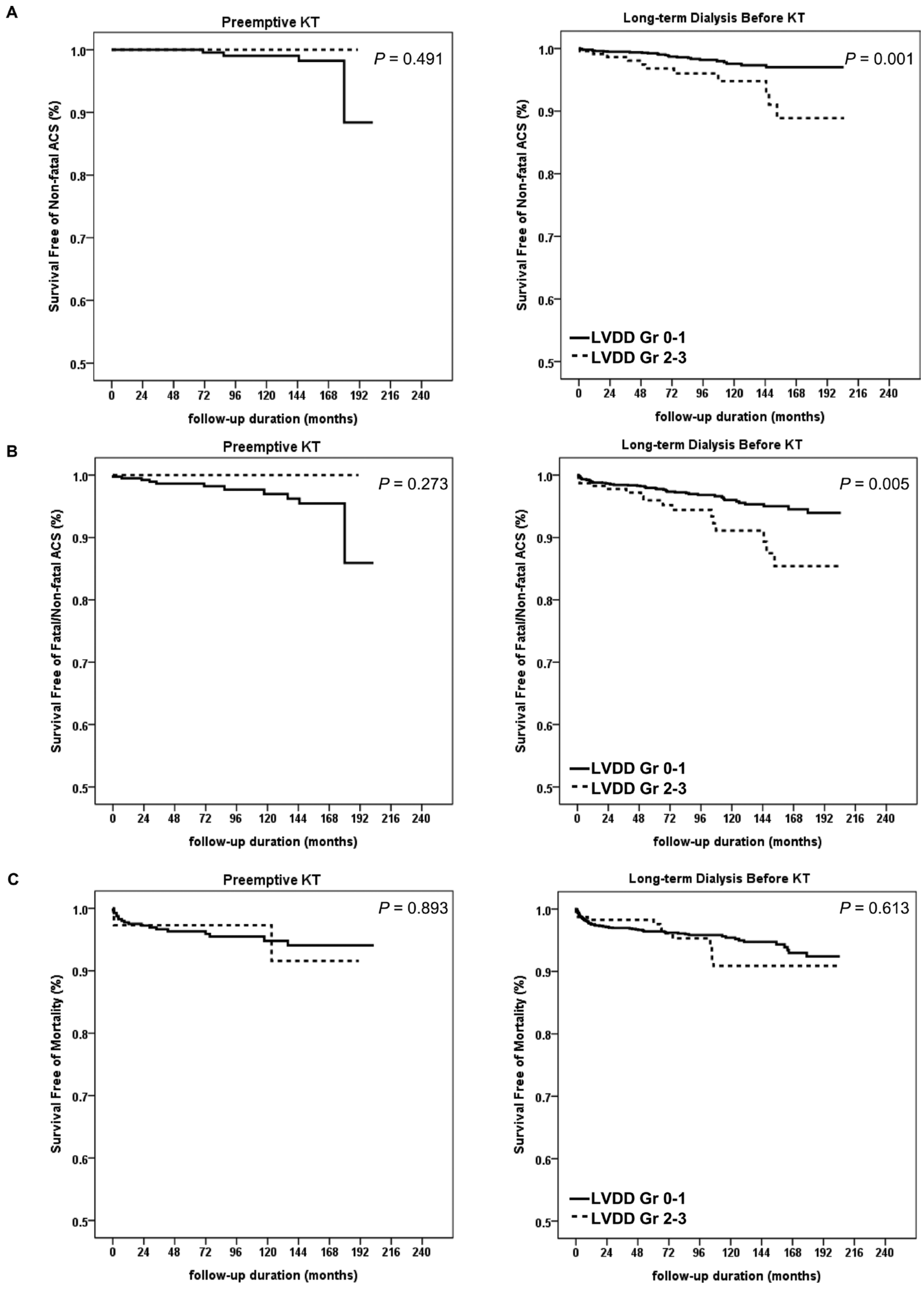

Supplementary Figure 1: Comparison of outcomes in patients with preemptive KT and patients with long-term dialysis before KT. Kaplan–Meier curves for posttransplant occurrence of non-fatal ACS (**A**), fatal/non-fatal ACS (**B**) and all-cause mortality (**C**) in the LVDD grade–based groups.

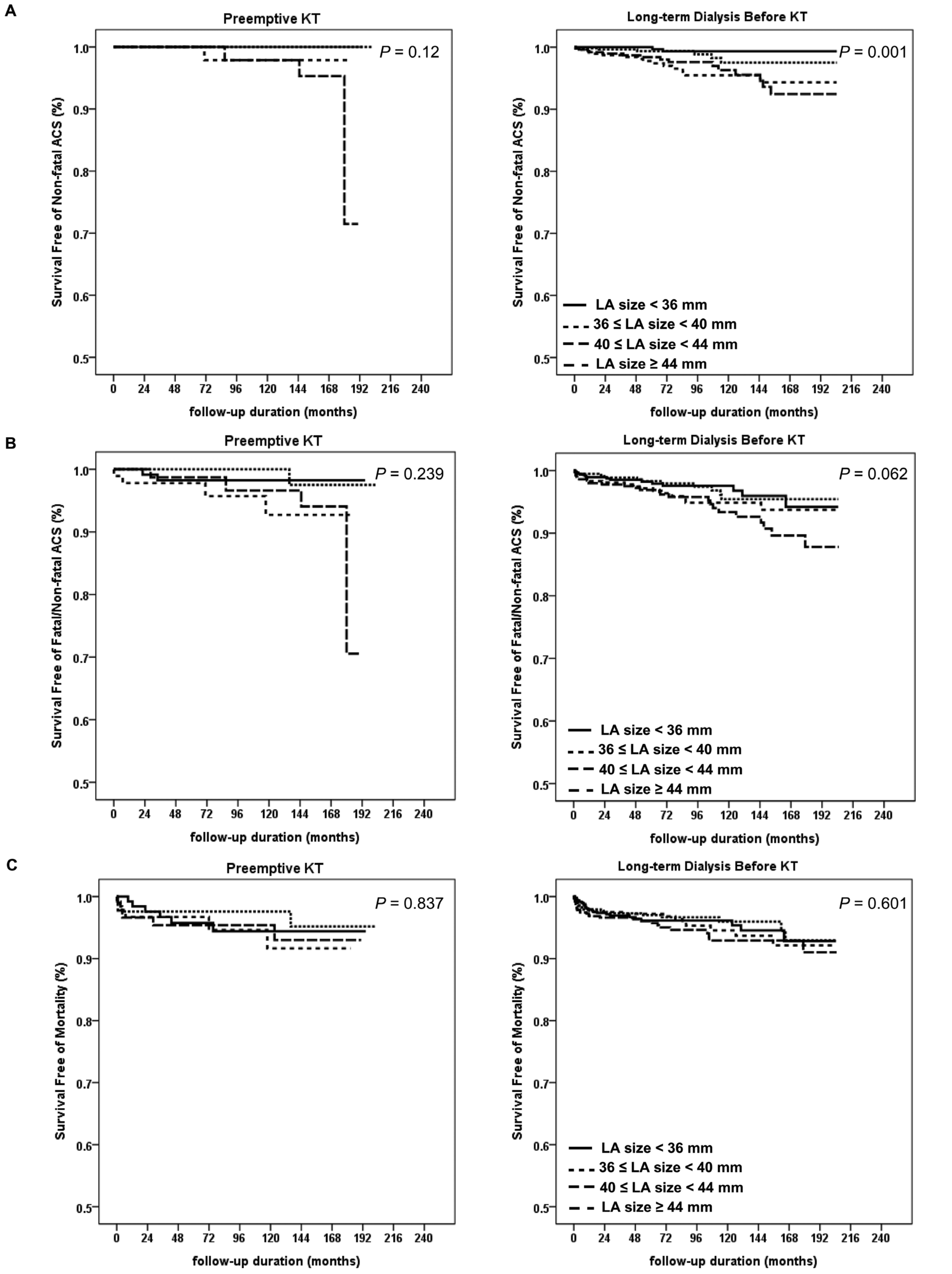

Supplementary Figure 2: Comparison of outcomes in patients with preemptive KT and patients with long-term dialysis before KT. Kaplan–Meier curves for posttransplant occurrence of non-fatal ACS (**A**), fatal/non-fatal ACS (**B**) and all-cause mortality (**C**) in the LA size–based groups.

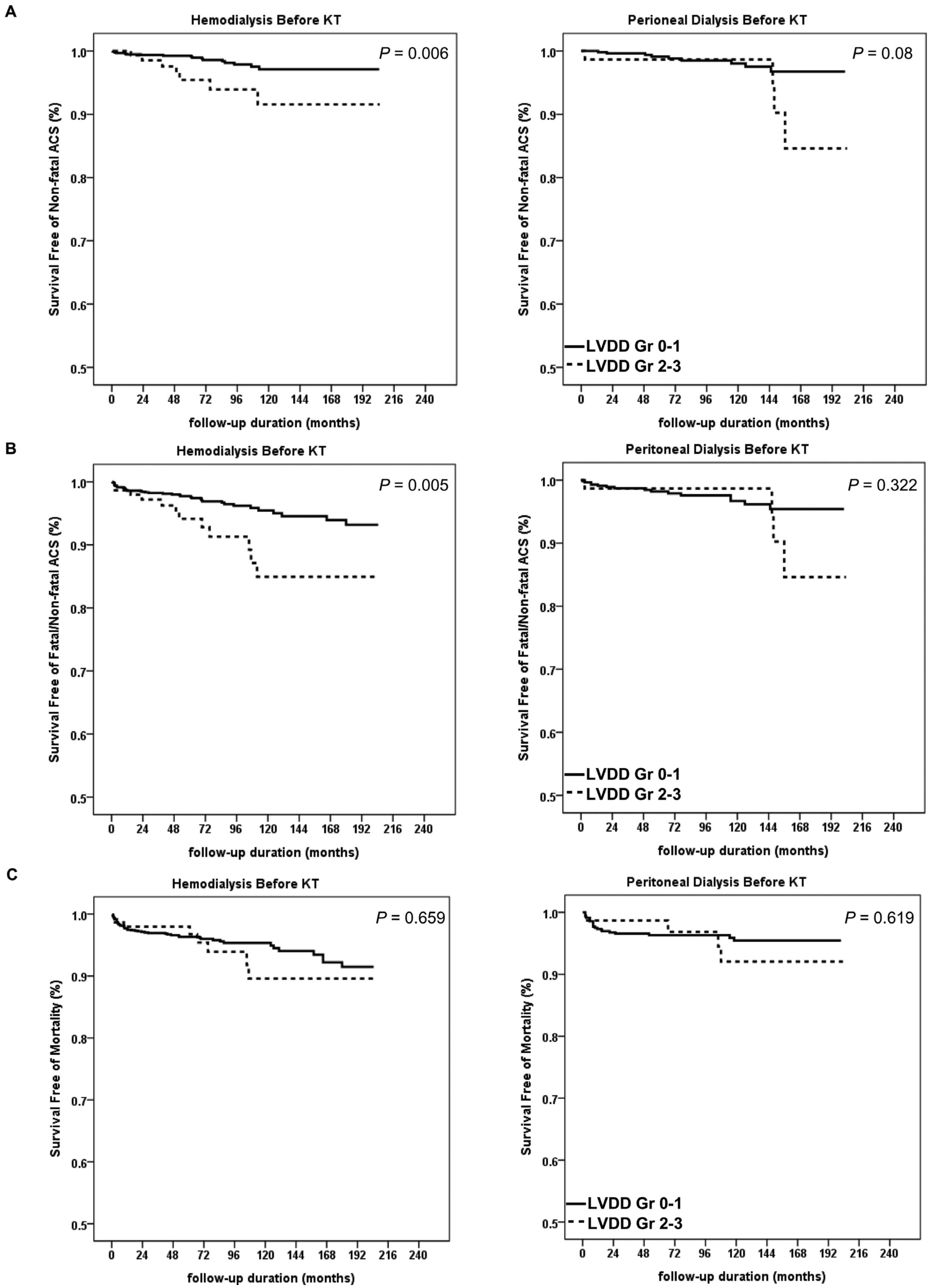

Supplementary Figure 3: Comparison of outcomes in patients with hemodialysis and peritoneal dialysis before KT. Kaplan–Meier curves for posttransplant occurrence of non-fatal ACS (**A**), fatal/non-fatal ACS (**B**) and all-cause mortality (**C**) in the LVDD grade–based groups
